# Influence of Fruit Maturity at Harvest on the Intensity of Smoke Taint in Wine

**DOI:** 10.3390/molecules20058913

**Published:** 2015-05-18

**Authors:** Renata Ristic, Paul K. Boss, Kerry L. Wilkinson

**Affiliations:** 1 School of Agriculture, Food and Wine, University of Adelaide, PMB 1, Glen Osmond, SA 5064, Australia; E-Mail: renata.ristic@adelaide.edu.au; 2 CSIRO Agriculture, PMB 2, Glen Osmond, SA 5064, Australia; E-Mail: paul.boss@csiro.au

**Keywords:** berry maturity, descriptive analysis, GC-MS, harvest date, smoke taint, wine

## Abstract

Bushfire smoke can affect the composition and sensory properties of grapes and wines, in some cases leading to wines which exhibit undesirable “smoky”, “ashy” and “medicinal” characters. This study investigated the extent to which fruit maturity (*i.e.*, ripeness) influences the perception of smoke taint in wine. Two white grape varieties (Chardonnay and Sauvignon Blanc) and two red grape varieties (Merlot and Shiraz) were exposed to smoke under experimental conditions, at approximately seven days post-veraison. Fruit was then harvested at two levels of maturity: Harvest A, when total soluble solids were 16–20 °Brix, *i.e.*, the berry ripeness typically required for production of sparkling or light-bodied wines; and Harvest B, when total soluble solids were 22–25 °Brix, *i.e.*, the berry ripeness typically required for production of full-bodied wines. The intensity of smoke taint in resulting wines was found to be influenced by fruit maturity, but differed between grape varieties. Smoke-related sensory attributes were apparent in Sauvignon Blanc wine made from early-harvested fruit and in Chardonnay wine made from late-harvested fruit, only; whereas Merlot and Shiraz wines exhibited smoke taint irrespective of fruit maturity. Smoke-derived volatile phenols, and various alcohols, esters and acids, were also quantified to determine the impact of smoke exposure and fruit maturity respectively, on wine composition.

## 1. Introduction

The sensory attributes of wine, in particular wine aroma and flavor, play an important role in determining our perception of wine quality and our preferences for different wine styles. Not surprisingly, considerable research has therefore been undertaken to understand the origins and contributions of the volatile compounds responsible for wine aroma and flavor. Many of these volatiles are known to accumulate in grapes in precursor forms, *i.e.*, as glycoconjugates, amino acid conjugates or fatty acids, and during fermentation and/or ageing, these precursors can be metabolized by yeast or enzymes, or hydrolyzed by acid to release the volatiles into wine [1].

Unfortunately, volatile compounds don’t always make a positive contribution to wine; occasionally, volatiles arise from spoilage or contamination and contribute off-aromas and flavors. Smoke taint provides one such example. Smoke from bushfires or prescribed burns can affect the composition and sensory properties of grapes and wine, in some cases leading to wines which exhibit undesirable “smoky”, “ashy” and “medicinal” characters, *i.e.*, smoke taint. The link between grape exposure to smoke and an apparent taint in wine was first demonstrated following post-harvest exposure of Verdelho grapes to smoke [2]. Several volatile phenols, including guaiacol, 4-methylguaiacol, syringol, 4-methylsyringol and *p*-, *m*- and *o*-cresols, have been identified in smoke-affected grapes and wine [2,3,4]; but more recently, the accumulation of smoke-derived volatile phenols in grapes and/or leaves in glycoconjugate forms has been demonstrated [4,5,6]. Metabolism of these glycoconjugates during fermentation can release the volatile phenols into wine [7,8] and smoke taint evolves in the form of unpalatable “smoky” and “ashy” aromas and flavors [7,9,10]. The timing and duration of grapevine exposure to smoke strongly influences the degree of smoke taint [11,12]; but winemaking techniques, in particular the duration of skin contact, and to a lesser extent, yeast strain selection and the use of oak and tannin additives, have also been shown to affect the intensity of smoke taint in wine [9]. Several methods for the amelioration of smoke tainted wine involving reverse osmosis and solid phase adsorption [13] or commercial fining agents [14] have also been reported.

The compositional changes that occur during berry maturation, *i.e.*, the decline in acid concentrations and the accumulation of sugars, varietal aroma and flavor (in free and precursor forms), and color and tannin (for red grape varieties), are well established [1]. This allows winemakers to make harvest decisions that optimize fruit composition for specific wine styles. Accordingly, fruit destined for light-bodied or sparkling wine styles will usually be harvested earlier (*i.e.*, at lower total soluble solids), than fruit destined for full-bodied wine styles which will be harvested later (and at higher total soluble solids). Since varietal characters develop with ripening, fruit maturity will influence the aroma and flavor profiles of both grapes and wine, but the impact of grapevine exposure to smoke on aroma and flavor development has not previously been considered; albeit the accumulation of guaiacol glycoconjugates in Merlot and Viognier fruit following grapevine exposure to smoke has been reported [6]. This study therefore aimed to investigate the extent to which fruit maturity (*i.e.*, ripeness) influences the perception of smoke taint in wine.

## 2. Results and Discussion

The composition and sensory properties of wines made with fruit harvested from control and smoke-affected Chardonnay, Sauvignon Blanc, Merlot and Shiraz grapevines, at two distinct maturity levels (*i.e.*, 16–20 °Brix for Harvest A *vs.* 22–25 °Brix for Harvest B), were determined in order to evaluate the influence of fruit maturity on the intensity of smoke taint.

### 2.1. Influence of Smoke Exposure and Fruit Maturity on Composition of Grapes and Wine

As intended, Harvest A fruit had significantly lower total soluble solids (TSS) levels than Harvest B fruit (Table 1), but significant differences in TSS were not observed between control and smoke-affected fruit from the same grape variety; *i.e.*, smoke exposure did not influence sugar accumulation. The average berry weight of Chardonnay, Merlot and Shiraz fruit was not affected by either fruit maturity or smoke exposure, but Sauvignon Blanc fruit had lower berry weight at Harvest A, compared to Harvest B.

Several differences in wine composition attributable to fruit maturity were also observed (Table 1). As expected, Harvest A wines had significantly lower levels of alcohol than their corresponding Harvest B wines. Harvest A white wines also exhibited lower pH, higher titratable acidity (TA) and lower total phenolics than Harvest B white wines. In some cases, significant differences were also observed between control and smoke-affected wines, e.g., alcohol content differed by up to 1.1% alcohol by volume (abv), while total phenolics were higher in smoke-affected Chardonnay and Sauvignon Blanc wines. While differences in pH were considered to be negligible, it was recognized that differences in TA could lead to perceptible differences in sensory ratings for acidity. Harvest A red wines contained significantly lower total phenolics than Harvest B red wines, but there was no difference attributable to grapevine exposure to smoke. The color density of red wines was also influenced by fruit maturity, with increased color density observed in Harvest B wines; but wine hue was consistent, irrespective of fruit maturity or smoke exposure. Similar pH and TA measurements were obtained for Merlot and Shiraz wines. Significant differences were observed in volatile acidity (VA) measurements, according to both fruit maturity and smoke exposure; however, VA levels were well below the maximum level (being 1.5 g/L) permitted in Australian wines [1] and were thus considered unlikely to impact on sensory analyses.

The proline content of wine was also measured (Table 1), as a potential indicator of environmental stress [15]. Proline concentrations ranged from 203 to 764 g/L in Harvest A wines and from 536 to 1807 g/L in Harvest B wines; in agreement with previous research that suggests proline accumulates in grapes with ripening [16,17]. Although significantly different proline levels were observed in control and smoke-affected Harvest B Shiraz wines, *i.e.*, 971 and 687 g/L respectively, grapevine exposure to smoke did not influence the proline content of any other wines (irrespective of fruit maturity).

**Table 1:** Berry weight and TSS of control and smoke-affected Chardonnay, Sauvignon Blanc, Merlot and Shiraz grapes, and color density, hue, total phenolics, proline, pH, titratable acidity (TA), volatile acidity (VA) and alcohol content of corresponding wines.

	Chardonnay				Sauvignon Blanc				Merlot				Shiraz			
	Harvest A		Harvest B		Harvest A		Harvest B		Harvest A		Harvest B		Harvest A		Harvest B	
	C	S	C	S	C	S	C	S	C	S	C	S	C	S	C	S
berry weight (g)	0.86	1.04	1.03	1.00	1.06 ^b^	1.14 ^b^	1.29 ^a^	1.28 ^a^	1.42	1.34	1.38	1.39	1.34	1.42	1.23	1.37
berry TSS (°Brix)	17.4 ^b^	18.2 ^b^	24.9 ^a^	25.5 ^a^	14.5 ^b^	16.0 ^b^	24.3 ^a^	24.4 ^a^	17.8 ^b^	18.2 ^b^	22.3 ^a^	22.2 ^a^	19.7 ^b^	19.6 ^b^	25.1 ^a^	24.3 ^a^
wine color density	-	-	-	-	-	-	-	-	1.9 ^b^	2.4 ^b^	3.9 ^a^	4.1 ^a^	4.1 ^b^	4.2 ^b^	8.4 ^a^	7.4 ^a^
wine hue	-	-	-	-	-	-	-	-	0.60	0.57	0.58	0.58	0.56	0.57	0.53	0.55
total phenolics (au)	1.5 ^d^	2.3 ^c^	3.0 ^b^	5.9 ^a^	1.4 ^c^	1.6 ^c^	2.4 ^b^	3.1 ^a^	13.4 ^b^	15.5 ^b^	19.7 ^a^	21.4 ^a^	19.2 ^b^	21.9 ^b^	36.7 ^a^	32.2 ^a^
pH	3.0 ^c^	2.9 ^d^	3.2 ^a^	3.2 ^b^	2.4 ^c^	2.6 ^b^	3.1 ^a^	3.2 ^a^	3.3	3.3	3.3	3.3	3.5 ^a^	3.3 ^b^	3.3 ^bc^	3.3 ^c^
TA ^**†**^ (g/L)	7.1 ^b^	8.6 ^a^	6.2 ^c^	8.6 ^a^	12.3 ^a^	10.6 ^b^	6.8 ^c^	6.5 ^c^	7.1 ^a^	6.8 ^ab^	6.4 ^c^	6.5 ^bc^	6.6 ^c^	7.3 ^b^	7.9 ^a^	8.0 ^a^
VA ^**†**^ (g/L)	0.14 ^c^	0.24 ^a^	0.18 ^b^	0.22 ^a^	0.45 ^a^	0.33 ^b^	0.26 ^c^	0.27 ^c^	0.18	0.17	0.19	0.17	0.15 ^c^	0.20 ^b^	0.30 ^a^	0.31 ^a^
alcohol (% abv)	10.4 ^c^	10.3 ^c^	13.0 ^a^	11.9 ^b^	8.0 ^d^	9.0 ^c^	14.7 ^b^	15.6 ^a^	9.5 ^b^	9.7 ^b^	13.0 ^a^	12.9 ^a^	10.7 ^b^	10.9 ^b^	13.7 ^a^	13.3 ^a^
proline (g/L)	315 ^b^	493 ^b^	1092 ^a^	1303 ^a^	203 ^b^	305 ^b^	536 ^a^	657 ^a^	411 ^b^	764 ^b^	1574 ^a^	1807 ^a^	299 ^c^	228 ^c^	971 ^a^	687 ^b^

Values represent the mean of three replicates. Values followed by different letters are significantly different (compared within each variety), *p* < 0.05; C = control; S = smoke-affected; **^†^**: TA and VA measured as tartaric acid and acetic acid equivalents respectively.

The concentrations of a range of smoke-derived volatile phenols were quantified in order to assess the extent to which wines were tainted by smoke (Table 2). None of the volatile phenols analyzed as markers of smoke taint were detected in control Chardonnay or Sauvignon Blanc wines. In contrast, guaiacol, syringol and cresols were detected at levels up to 3 µg/L in control Merlot wines and at higher levels, *i.e.*, 2 to 11 µg/L, in control Shiraz wines; but this was consistent with previous studies which have shown guaiacol and 4-methylguaiacol can occur naturally in fruit of some red grape varieties [18,19]. Comparatively higher volatile phenol concentrations were detected in wines made from smoke-affected fruit. Smoke-affected Chardonnay wines contained up to 3 and 5 µg/L of guaiacol and syringol, respectively; while smoke-affected Sauvignon Blanc wines contained slightly higher levels, *i.e.*, up to 4 and 6 µg/L, respectively. 4-Methylguaiacol and cresols were also detected in wine made from smoke-affected Sauvignon Blanc fruit (from Harvest A). Elevated concentrations of the smoke-derived volatile phenols were observed in smoke-affected Merlot and/or Shiraz wines, with guaiacol and syringol the most abundant volatile phenols, at concentrations of 8 to 15 and 18 to 28 µg/L, respectively. This is consistent with previous research which demonstrated grape skins contained higher proportions of guaiacol glycoconjugates (by mass) than pulp [6]. The inclusion of skins during red winemaking therefore allows for greater extraction of smoke-derived volatile phenols and their precursors. Indeed, the duration of skin contact has been found to influence volatile phenol levels in smoke tainted wines [9]. The highest volatile phenol levels were observed in smoke-affected Harvest B Shiraz wine, but taking into account the volatile phenol content of control wines, compositional data suggested smoke-affected Merlot wines were likely to be more heavily tainted than smoke-affected Shiraz wines. With the exception of guaiacol, most volatile phenols occurred at concentrations below their reported detection thresholds, being: 9.5 and 570 µg/L for guaiacol and syringol respectively, in 10% model wine [20], 21 µg/L for 4-methylguaiacol in water [21], 100,000 µg/L for 4-methylsyringol in water [22], and between 10 and 68 µg/L for *p*-, *m*- and *o*-cresols in 10% aqueous ethanol [23]. A recent study suggests a combination of volatile phenols and their glycoconjugate precursors contribute to the unpalatable aromas and flavors of smoke tainted wines [24]; with the “ashy aftertaste” thought to be associated with in-mouth release of volatile phenols from glycoconjugates [25]. Glycoconjugates were not measured in the current study; instead sensory analysis was performed to determine the intensity of a range of sensory attributes, including smoke-related aromas and flavors, for each wine.

**Table 2:** Concentrations (µg/L) of smoke-derived volatile phenols in control and smoke-affected Chardonnay, Sauvignon Blanc, Merlot and Shiraz wines.

	Chardonnay				Sauvignon Blanc				Merlot				Shiraz			
	Harvest A		Harvest B		Harvest A		Harvest B		Harvest A		Harvest B		Harvest A		Harvest B	
	C	S	C	S	C	S	C	S	C	S	C	S	C	S	C	S
guaiacol	nd	3	nd	2	nd	4	nd	3	tr	21 ^a^	1 ^b^	18 ^a^	8 ^c^	22 ^b^	11 ^c^	28 ^a^
4-methylguaiacol	nd	tr	nd	tr	nd	1	nd	nd	nd	6 ^a^	nd	5 ^a^	nd	3 ^a^	nd	3 ^a^
syringol	nd	5 ^a^	nd	2 ^b^	nd	6 ^a^	nd	3 ^b^	2 ^c^	15 ^a^	3 ^c^	9 ^b^	5 ^b^	8 ^a^	6 ^b^	9 ^a^
4-methylsyringol	nd	nd	nd	nd	nd	nd	nd	nd	nd	3 ^a^	nd	3 ^a^	nd	tr	nd	nd
total cresols	nd	nd	nd	nd	nd	6	nd	nd	tr	4 ^a^	tr	5 ^a^	2 ^c^	4 ^b^	3 ^c^	6 ^a^

Values represent the mean of three replicates. Values followed by different letters are significantly different (compared within each variety), *p* < 0.05; nd = not detected; tr = trace (*i.e.*, positive identification, but <1 µg/L); C = control; S = smoke-affected.

### 2.2. Influence of Smoke Exposure and Fruit Maturity on Wine Sensory Properties

**Figure 1:**
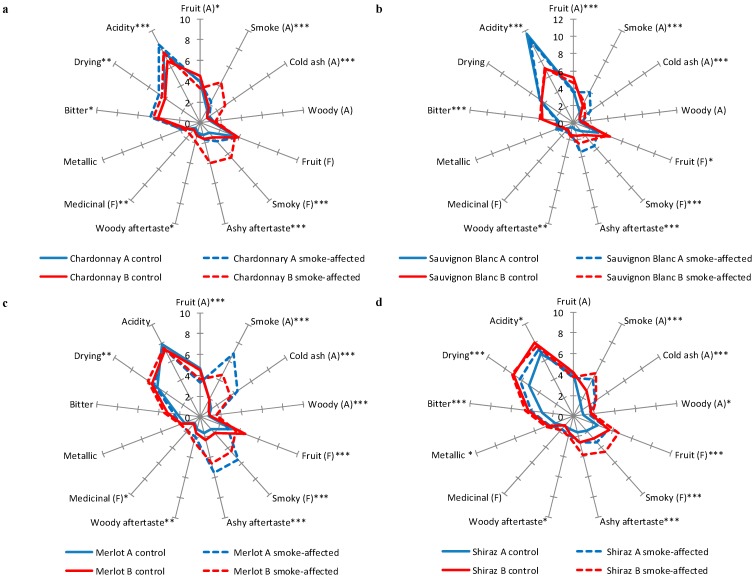
Mean ratings for sensory attributes of (**a**) Chardonnay (**b**) Sauvignon Blanc (**c**) Merlot and (**d**) Shiraz wines, made from control and smoke-affected grapes harvested at two different levels of maturity; A = aroma; F = flavor; (*: *p* < 0.05; **: *p* < 0.01, ***: *p* < 0.001).

Wines made from control and smoke-affected fruit were subjected to descriptive analysis using a trained sensory panel. Panelists rated the intensity of various aroma and palate attributes (Table 3), to determine the sensory profile of each wine (Figure 1, Supplementary Table S1); with significant differences observed between Harvest A and B wines and/or control and smoke-affected wines (compared within each variety). The intensity of fruit aromas and flavors of Harvest B wines were perceived to be equal to, or higher than Harvest A wines, demonstrating the influence of maturity at the time of harvest, on the intensity of fruit expression. Acidity ratings ranged from 6.7 to 8.5, except for Harvest A Sauvignon Blanc wines, which were given ratings of 10.7 and 11.6. These wines were considered to be significantly more acidic (Figure 1b), reflecting their high TA (Table 1).

**Table 3:** Aroma and palate attributes used for sensory analysis of control and smoke-affected wines.

Attribute	Description
*Aroma*	
Fruit	The overall intensity of fruit aroma
Smoke	Perception of smoke aroma, including smoked meat/bacon, toasty, charry, cigar box
Cold ash	Burnt aroma associated with ash, including ashtray, tarry, campfire
Earthy	Aromas associated with musty, dusty, wet wood, barnyard, mushroom, dank, moldy
Medicinal	Aromas characteristic of band-aids, disinfectant, cleaning products, solvents
*Palate*	
Fruit flavor	The overall intensity of fruit flavor
Smoky flavor	Perception of smoke flavor, including bacon and smoked meat
Ashy aftertaste	Length of taste associated with residue of ashtray perceived in the mouth after expectorating, including coal ash, ashtray, tarry, acrid, campfire
Woody aftertaste	Length of taste associated with woody residue, including wood, oak, pencil shavings
Medicinal flavor	Flavors characteristic of band-aids, disinfectant, cleaning products, solvents
Metallic	The “tinny” flavor associated with metals
Bitter	Intensity of bitter taste/aftertaste
Drying	Drying, puckering mouthfeel after expectoration of the wine
Acidity	Intensity of sour/acid taste

The sensory profiles of Harvest A Chardonnay wines (Figure 1a) were surprisingly similar; indeed acidity was the only attribute perceived to be significantly different and there were no apparent smoke-related aromas or flavors evident. In contrast, significant differences were perceived in the sensory attributes of Harvest B Chardonnay wines; with noticeable “smoke”, “cold ash” and “woody” aromas, “smoky” flavor and an “ashy aftertaste” observed in the Harvest B smoke-affected Chardonnay wine. Despite slightly higher levels of guaiacol and syringol being detected in the Harvest A smoke-affected Chardonnay wine, the corresponding Harvest B wine was considered to be more heavily tainted, based on sensory analysis results. In the case of Sauvignon Blanc wines, smoke-related sensory attributes were perceived in both of the wines made from smoke-affected fruit (Figure 1b). The Harvest A smoke-affected Sauvignon Blanc wine exhibited “smoke” and “cold ash” aromas, “smoky” flavor and an “ashy aftertaste”; while the corresponding Harvest B wine exhibited “smoky” flavor and “ashy aftertaste” only. The somewhat diminished smoke attributes observed in this wine might have resulted from the enhanced fruit aromas and flavors derived from the riper Harvest B fruit; similar observations were made in a previous study that investigated the effect of defoliation on the intensity of smoke taint in Chardonnay wines [10].

Compared to smoke-affected white wines, smoke-affected red wines were found to exhibit considerably more apparent smoke-related aromas and flavors, in agreement with the compositional data (Table 2); which can largely be attributed to the increased duration of skin contact associated with red winemaking, and therefore increased extraction of smoke-derived volatile phenols and their glycoconjugates [9]. However, fruit maturity also influenced the intensity of smoke taint, with significant differences observed between the sensory profiles of smoke-affected Harvest A *vs.* Harvest B wines. Smoke-affected Merlot wines exhibited intense “smoke” and “cold ash” aromas, “smoky” and “medicinal” flavors and “ashy aftertaste”, and diminished “fruit” aroma and flavor (Figure 1c); whereas smoke-affected Shiraz wines exhibited moderate “smoke” aroma and in the case of the Harvest B smoke-affected Shiraz wine, moderate “smoky” flavor and “ashy aftertaste”, despite significantly higher “fruit” flavor (Figure 1d). Sensory data showed good agreement with compositional data; with descriptive analysis confirming Harvest A smoke-affected Sauvignon Blanc to be the most tainted white wine and Harvest A smoke-affected Merlot to be the most tainted red wine.

The lack of strong sensory evidence for smoke taint in the early harvest Chardonnay wine has implications for the use of such techniques for predicting the impact of smoke on grapes at harvest, as it demonstrates a “false negative” result. Furthermore, the lower scores for smoke-related attributes in Sauvignon Blanc wines suggest that “false positives” may also be possible when early harvested fruit is used to predict harvest quality. While false positives may be less of a concern since winemakers would likely err on the side of caution, they constitute a serious issue for affected grape growers. False negatives are of more concern to winemakers, given the costs associated with producing wine which is later found to be tainted. In the future, it will be important to identify those varieties that behave like Chardonnay with regards to the evolution of smoke-derived sensory attributes, and to then develop better predictive methods to assist in harvest decisions. This may involve developing a better understanding of the dynamics of the glycosylated volatile phenol pool, to see if subtle changes in the number and type of sugar moieties present could explain the developmentally driven changes in sensory results. It may also be better to evaluate test wines from early harvested white fruit, produced according to red winemaking practices, *i.e.*, with skins present during fermentation, to eliminate the risk of false negatives; albeit the incidence of false positives may increase. Alternatively, determination of glycoconjugates may provide more reliable evaluations. However this requires access to expensive equipment and sensitive analytical methodologies. Insight into the sensory interactions of smoke-derived volatile compounds with other wine volatiles may also afford strategies for optimizing the concentrations of enhancers and suppressors of smoke volatiles, to reduce their sensory impact.

### 2.3. Influence of Smoke Exposure and Fruit Maturity on Grape and Yeast-Derived Wine Volatiles

Non-targeted headspace analysis of control and smoke-affected wines was performed to determine the impact of smoke exposure and fruit maturity on the concentrations of several grape and/or yeast-derived wine volatiles. Previous research has demonstrated that the date of harvest and/or the TSS of fruit can significantly influence the volatile composition of wine [26], but to date, any potential impact of smoke exposure on grapevine secondary metabolism has not been considered. In the current study, 20 alcohols, esters and acids were quantified (Table 4), relative to an internal standard (*i.e.*, *d_13_*-hexanol). These compounds were not intended to be a comprehensive representation of the volatile composition of the different wines studied, since this would require quantification of a much broader range of volatiles, so as to adequately reflect varietal expression. Rather, this analysis was undertaken to determine whether or not grapevine exposure to smoke impacted on wine composition, beyond the occurrence of smoke-derived volatile phenols.

**Table 4:** Relative peak areas of selected volatiles detected in control and smoke-affected Chardonnay, Sauvignon Blanc, Merlot and Shiraz wines.

Volatile Compounds (Aroma Descriptors [27 and References Therein])	Chardonnay				Sauvignon Blanc				Merlot				Shiraz			
	Harvest A		Harvest B		Harvest A		Harvest B		Harvest A		Harvest B		Harvest A		Harvest B	
	C	S	C	S	C	S	C	S	C	S	C	S	C	S	C	S
*trans*-3-hexen-1-ol (grassy)	12	10	19	14	23 ^c^	69 ^a^	42 ^b^	18 ^c^	tr	tr	tr	tr	323 ^a^	192 ^b^	144 ^b^	53 ^c^
*cis*-3-hexen-1-ol (green)	14	16	8	11	145 ^a^	144 ^a^	19 ^b^	18 ^b^	tr	tr	tr	tr	113 ^b^	148 ^a^	72 ^c^	69 ^c^
2-phenylethanol (rose)	2429	3416	6633	3657	4178 ^b^	8170 ^b^	12,519 ^a^	12,613 ^a^	4379 ^c^	4964 ^bc^	6374 ^ab^	7322 ^a^	14,892 ^b^	16,012 ^b^	24,075 ^a^	20,569 ^a^
isoamyl alcohol (solvent)	3561 ^b^	3635 ^b^	4795 ^a^	4365 ^a^	8757 ^d^	10,347^c^	16,762 ^b^	18,001 ^a^	4595 ^b^	4671 ^b^	6562 ^a^	6159 ^a^	15,357	16,941	18,379	18,567
isobutyl acetate (banana, pear)	254 ^d^	290 ^c^	361 ^b^	442 ^a^	55 ^d^	97 ^c^	248 ^b^	332 ^a^	162 ^a^	178 ^a^	103 ^b^	98 ^b^	nd	nd	nd	nd
2-phenylethyl acetate (rosewater)	128	196	283	240	154 ^c^	260 ^c^	966 ^b^	1157 ^a^	25	20	27	28	45	51	56	43
diethyl succinate (caramel)	394 ^c^	397 ^c^	727 ^b^	1148 ^a^	538 ^b^	749 ^b^	2323 ^a^	2416 ^a^	414 ^b^	475 ^b^	1071 ^a^	1044 ^a^	1431 ^b^	1548 ^b^	2994 ^a^	2828 ^a^
ethyl 2-methyl butanoate (berry)	23 ^b^	22 ^b^	39 ^a^	31 ^ab^	66 ^c^	73 ^c^	85 ^b^	95 ^a^	43 ^b^	51 ^b^	82 ^a^	104 ^a^	180 ^b^	188 ^b^	329 ^a^	244 ^ab^
ethyl butanoate (fruity, strawberry)	326	271	345	300	943 ^b^	979 ^b^	1813 ^a^	1747 ^a^	116 ^b^	127 ^b^	174 ^a^	171 ^a^	333 ^b^	325 ^b^	540 ^a^	152 ^c^
ethyl 3-methylbutanoate (red fruit)	49 ^c^	112 ^a^	65 ^b^	48 ^c^	103 ^c^	112 ^c^	166 ^b^	185 ^a^	79 ^b^	78 ^b^	79 ^b^	122 ^a^	201 ^b^	249 ^b^	245 ^b^	402 ^a^
isoamyl acetate (banana)	4261 ^c^	5174 ^b^	3519 ^d^	7613 ^a^	8504 ^d^	12,504 ^c^	33,395 ^b^	40,277 ^a^	304 ^c^	675 ^b^	935 ^a^	667 ^b^	4667 ^a^	3873 ^b^	4852 ^a^	4372 ^a^
ethyl phenylacetate (floral)	70	21	40	26	49	55	66	66	28 ^c^	32 ^bc^	49 ^ab^	63 ^a^	117 ^c^	120 ^c^	260 ^a^	201 ^b^
*trans*-3-hexen-1-ol acetate (fruity)	35 ^b^	50 ^a^	17 ^b^	25 ^ab^	141 ^b^	318 ^a^	67 ^c^	82 ^c^	tr	tr	tr	tr	29	19	12	14
*cis*-3-hexen-1-ol acetate (fruity)	40 ^b^	37 ^b^	61 ^a^	9 ^c^	68 ^c^	192 ^bc^	207 ^b^	373 ^a^	tr	tr	tr	tr	tr	tr	tr	tr
ethyl acetate (nail polish)	248 ^b^	286 ^ab^	358 ^ab^	435 ^a^	490 ^b^	653 ^b^	1991 ^a^	2174 ^a^	215 ^b^	212 ^b^	319 ^a^	311 ^a^	770 ^b^	691 ^b^	1171 ^a^	1117 ^a^
ethyl hexanoate (green apple)	7760	7607	8087	5674	40 ^bc^	37 ^c^	47 ^b^	63 ^a^	2267 ^b^	2611 ^b^	3654 ^a^	2509 ^b^	8227	7574	9007	8318
ethyl octanoate (fruity)	20,348	21,380	22,801	19,213	20,653 ^b^	24,149 ^b^	41,157 ^a^	40,783 ^a^	3606 ^b^	4070 ^b^	7304 ^a^	6124 ^a^	8922 ^b^	8850 ^b^	11,432 ^a^	10,105 ^ab^
hexanoic acid (sweaty)	1448	1164	1202	836	4050 ^a^	3586 ^a^	3268 ^ab^	2482 ^b^	280	320	356	332	315 ^b^	344 ^b^	671 ^a^	503 ^ab^
octanoic acid (rancid cheese)	4386	3860	3908	3277	14,083	13,729	14,315	13,236	404	457	556	460	536 ^a^	558 ^ab^	239 ^bc^	181 ^c^
decanoic acid (plasticine)	2576	1978	1994	1394	6788	7345	6271	5615	tr	tr	tr	tr	16	21	10	7

Values represent the mean of three replicates. Values followed by different letters are significantly different (compared within each variety), *p* < 0.05; nd = not detected; tr = trace (*i.e.*, positive identification only); C = control; S = smoke-affected.

Most of the compounds quantified were esters (Table 4), which were likely formed by yeast during fermentation [27]. Some volatiles were detected in certain varieties only, e.g., the isomers of 3-hexen-1-ol and 3-hexen-1-ol acetate, which provides further evidence for the influence of grape composition on the production of some wine esters [28,29]. The relative abundance of several volatiles, e.g., isobutyl alcohol, ethyl butanoate, ethyl 2-methylbutanoate and 2-phenylethyl acetate, differed significantly between Harvest A and B wines. For example, this was particularly evident when Sauvignon Blanc wines were compared by fruit maturity (Table 4), and may account for the decreased intensity of smoke-related sensory attributes observed in smoke-affected Sauvignon Blanc wine at Harvest B, compared to Harvest A (Figure 1b). In contrast, higher ester concentrations in Harvest B Shiraz wines (compared to Harvest A Shiraz wines) did not have the same sensory impact, as smoke-affected Shiraz wines had notably higher ratings for smoke-related attributes compared to the corresponding wine produced from Harvest A fruit (Figure 1d). There were no major differences in the volatile profiles of the early-harvested Chardonnay wines and those produced from grapes harvested later that would have suggested there was suppression of smoke-related sensory attributes in the early-harvest Chardonnay wines. In some cases, compositional differences were also observed between control and smoke-affected wines, e.g., 2-phenylethyl acetate, ethyl hexanoate and isoamyl acetate; albeit trends directly attributable to grapevine smoke exposure were not apparent. Nonetheless, these results suggest smoke exposure might influence the accumulation of some grape-derived volatile compounds and/or their precursors. Future research could therefore investigate to what extent smoke exposure affects grape secondary metabolism.

## 3. Experimental Section

### 3.1. Field Application of Smoke to Grapevines

Chardonnay, Sauvignon Blanc, Merlot and Shiraz grapevines growing in vineyards located at the University of Adelaide’s Waite Campus in Adelaide, South Australia (34°58′S, 138°38′E) were enclosed in purpose-built smoke tents and exposed to smoke for 1 h, at approximately 7 days post-veraison, under experimental conditions described previously [7]. Control (*i.e.*, unsmoked grapevines) and smoke treatments (each in triplicate, with three vines per replicate per variety) were established along a single row (for each variety), with buffers (comprising at least 3 grapevines) between treatments and replicates.

### 3.2. Winemaking

Fruit was harvested at two distinct time points, being: (i) Harvest A, when TSS was 16–20 °Brix, *i.e.*, the berry ripeness typically required for production of sparkling or light-bodied wines; and (ii) Harvest B, when TSS was 22–25 °Brix, *i.e.*, the berry ripeness typically required for production of full-bodied wines. For Chardonnay and Sauvignon Blanc, fruit was stored overnight in a 3 °C cool room, then parcels comprising approximately 15 kg (per field replicate per treatment) were de-stemmed, crushed and pressed, with the addition of 50 mg/L of sulfur dioxide (added as an 8% solution of potassium metabisulphite (PMS)) and 0.01 g/L Lallzyme HC (Lallemand, Australia). Following the addition of diammonium phosphate (150 mg/L), juice was inoculated with 0.3 g/L EC-1118 yeast (Lallemand, Australia) and fermented at 10–12 °C until the residual sugar approached 0 g/L. Wines were then racked from gross lees and cold stabilized (at 0 °C for 3 months). For Merlot and Shiraz, fruit parcels of approximately 15 kg (per field replicate per treatment) were crushed, with the addition of 50 mg/L sulfur dioxide (as PMS). Following the addition of diammonium phosphate (200 mg/L), must was inoculated with 0.3 g/L EC-1118 yeast (Lallemand, Australia) and fermented on skins at 24 °C, with the cap plunged three times per day. When the residual sugar approached 0 g/L, wines were racked from gross lees and cold stabilized (at 0 °C for 3 months). Malolactic fermentation was not performed on white or red wines. Wine free SO_2_ was adjusted to 30 mg/L respectively, before bottling (under screw cap closures). Bottles were stored at 15 °C for six months prior to chemical and sensory analyses.

### 3.3. Chemical Analysis

Wine pH, titratable acidity (TA), volatile acidity (VA) and ethanol content (as % abv) were determined by the Australian Wine Research Institute’s (AWRI) Commercial Services Laboratory (Adelaide, Australia) with a FOSS FTIR WineScan. Wine color density, wine hue and total phenolics were determined according to previously described methods [30]. Proline was determined via the isatin method [31]. The concentrations of the volatile phenols, guaiacol, 4-methylguaiacol, syringol, 4-methylsyringol, and *p*-, *m*- and *o*-cresols (as total cresols), were determined by the AWRI’s Commercial Services Laboratory, using an Agilent 6890 gas chromatograph coupled to a 5973 mass selective detector (Agilent, Palo Alto, CA, USA), and stable isotope dilution analysis methods reported previously [4,32]. These publications describe the preparation of deuterated internal standards (*d_3_*-guaiacol, *d_3_*-4-methylguaiacol, *d_3_*-syringol and *d_7_*-*o*-cresol), method validation and instrumental operating conditions.

### 3.4. Non-Targeted Headspace Volatile Analysis

The volatile constituents of the wines produced from control and smoke-treated fruit were analyzed using an SPME-GC-MS method. The wines were analyzed at two different concentrations, 1 in 100 or 1 in 2 diluted with water to a final volume of 10 mL and 3 g of sodium chloride was added to each 20 mL vial prior to sample addition. The extraction and chromatographic conditions were identical to that described elsewhere [27]. The volatiles were identified by comparing mass spectra with those of authentic standards and spectral libraries. A laboratory generated library (328 compounds) as well as the US National Institute of Standards and Technology-11 (NIST-11) and the Wiley Registry 9th Edition mass spectral libraries were used for identification purposes. When compounds matched both the mass spectra and linear retention indices (LRI) of that of authentic standards they were considered positively identified. LRI was calculated from a compound’s retention time relative to the retention of a series of *n*-alkanes (C_8_–C_26_). Other compounds were tentatively identified based upon comparison with mass spectral libraries and published LRI, or comparisons with mass spectral libraries alone. ChemStation (Agilent) was used to quantify the components of the samples relative to the internal standard (*d_13_*-hexanol) using the peak area of an extracted ion. The extracted ions used for quantification have been listed elsewhere [33], except for *trans-*3-hexen-1-ol acetate and *cis-*3-hexen-1-ol acetate both of which were quantified using the 67 *m/z* ion.

### 3.5. Sensory Analysis

Descriptive sensory analysis of wines comprised a series of training sessions and formal evaluations (4 each), held twice weekly. A tasting panel comprising 10 female and 3 male staff and students from the University of Adelaide and the Australian Wine Research Institute was convened; with the exception of one panelist, the panel had extensive previous experience in descriptive analysis of smoke tainted wines. Prior to sensory analysis, each wine replicate was informally assessed by a panel of four experienced tasters, in order to assess any artefacts or off-flavors, and to determine the scope of differences between treatments. During the training sessions, the tasting panel generated appropriate descriptive terms (Table 3) and gained familiarity in recognizing and scoring the intensity of each attribute, following the procedure outlined by Lawless and Heymann [34]. Formal evaluations were then conducted in isolated booths at 22–23 °C under sodium lights during two consecutive sensory sessions for each variety; *i.e.*, with three wine replicates from each experimental treatment presented in each session. All wines were presented as 30 mL samples in 3-digit coded, covered, ISO standard wine glasses. The presentation order was randomized across panelists using Design Express (Qi Statistics and Product Perception, Berkshire, UK). Panelists rated aroma and palate attributes using a 15 cm unstructured line scale, with anchor points of “low” (at 10% of the line) and “high” (at 90% of the line). Panelists rinsed thoroughly with pectin solution (1 g/L) and water, and rested for at least 45 seconds between samples. Data acquisition was carried out using Fizz (Version 2.4, Biosystemes, Couternon, France).

### 3.6. Statistical Analysis

Chemical data were analyzed by analysis of variance (ANOVA) using JMP (Version 7, SAS Institute, Cary, NC, USA). Mean comparisons were performed by Tukey’s HSD post-hoc test at *p* < 0.05. Sensory data were analyzed using SenPaq (Version 4.82, Qi Statistics 2009) and a mixed model ANOVA performed to determine the effects of treatment, fermentation replicate, presentation replicate and judge (treating judges in a random effect). The method for discrimination of means was Fisher’s least significant difference at *p* < 0.1.

## 4. Conclusions

The perceived intensity of smoke taint in wines made from smoke-affected fruit was found to be influenced by fruit maturity, but differed between grape varieties. For white grape varieties, smoke-related sensory attributes were apparent in Sauvignon Blanc wine made from early harvested fruit, but for Chardonnay, only wines made from late harvested fruit were found to be tainted. In contrast, Merlot and Shiraz wines exhibited smoke taint irrespective of fruit maturity; albeit, the intensity of smoke aromas and flavors were rated higher in Merlot wine made from early harvested fruit and in Shiraz wine made from late harvested fruit. These findings have important implications for grape growers and winemakers with respect to assessing smoke taint, since standard practice following vineyard exposure to smoke typically involves sampling fruit as early as possible, conducting small-lot fermentations, and then analyzing the resulting wine (chemically and/or sensorially). It is clear from this study that this process could lead to “false negatives”, whereby the extent of smoke taint is either underestimated, or not detected at all. The absence of high levels of volatile phenols and/or perceptible smoke-related sensory attributes in wines made from early-harvested fruit, may not guarantee they have not been tainted by bushfire smoke; especially for white grape varieties.

## Supplementary Materials

Supplementary materials can be accessed at: http://www.mdpi.com/1420-3049/20/05/8913/s1.

## References

[1] Jeffery D.W., Wilkinson K.L., Bamforth C.W., Ward R.E. (2014). Wine. The Oxford Handbook of Food Fermentations.

[2] Kennison K.R., Wilkinson K.L., Williams H.G., Smith J.H., Gibberd M.R. (2007). Smoke-derived taint in wine: Effect of postharvest smoke exposure of grapes on the chemical composition and sensory characteristics of wine. J. Agric. Food Chem..

[3] Sheppard S.I., Dhesi M.K., Eggers N.J. (2009). Effect of pre- and post-veraison smoke exposure on guaiacol and 4-methylguaiacol concentration in mature grapes. Am. J. Enol. Vitic..

[4] Hayasaka Y., Baldock G.A., Parker M., Pardon K.H., Black C.A., Herderich M.J., Jeffery D.W. (2010). Glycosylation of smoke-derived volatile phenols in grapes as a consequence of grapevine exposure to bushfire smoke. J. Agric. Food Chem..

[5] Hayasaka Y., Dungey K.A., Baldock G.A., Kennison K.R., Wilkinson K.L. (2010). Identification of a β-d-glucopyranoside precursor to guaiacol in grape juice following grapevine exposure to smoke. Anal. Chim. Acta.

[6] Dungey K.A., Hayasaka Y., Wilkinson K.L. (2011). Quantitative analysis of glycoconjugate precursors of guaiacol in smoke-affected grapes using liquid chromatography-tandem mass spectrometry based stable isotope dilution analysis. Food Chem..

[7] Kennison K.R., Gibberd M.R., Pollnitz A.P., Wilkinson K.L. (2008). Smoke-derived taint in wine: The release of smoke-derived volatile phenols during fermentation of Merlot juice following grapevine exposure to smoke. J. Agric. Food Chem..

[8] Wilkinson K.L., Ristic R., Pinchbeck K.A., Fudge A.L., Singh D.P., Pitt K.M., Downey M.O., Baldock G.A., Hayasaka Y., Parker M. (2011). Comparison of methods for the analysis of smoke related phenols and their conjugates in grapes and wine. Aust. J. Grape Wine Res..

[9] Ristic R., Osidacz P., Pinchbeck K.A., Hayasaka Y., Fudge A.L., Wilkinson K.L. (2011). The effect of winemaking techniques on the intensity of smoke taint in wine. Aust. J. Grape Wine Res..

[10] Ristic R., Pinchbeck K.A., Fudge A.L., Hayasaka Y., Wilkinson K.L. (2013). Effect of leaf removal and grapevine smoke exposure on colour, chemical composition and sensory properties of Chardonnay wines. Aust. J. Grape Wine Res..

[11] Kennison K.R., Wilkinson K.L., Pollnitz A.P., Williams H.G., Gibberd M.R. (2009). Effect of timing and duration of grapevine exposure to smoke on the composition and sensory properties of wine. Aust. J. Grape Wine Res..

[12] Kennison K.R., Wilkinson K.L., Pollnitz A.P., Williams H.G., Gibberd M.R. (2011). Effect of smoke application to field-grown Merlot grapevines at key phenological growth stages on wine sensory and chemical properties. Aust. J. Grape Wine Res..

[13] Fudge A.L., Ristic R., Wollan D., Wilkinson K.L. (2011). Amelioration of smoke taint in wine by reverse osmosis and solid phase adsorption. Aust. J. Grape Wine Res..

[14] Fudge A.L., Schiettecatte M., Ristic R., Hayasaka Y., Wilkinson K.L. (2012). Amelioration of smoke taint in wine by treatment with commercial fining agents. Aust. J. Grape Wine Res..

[15] Mohammadkhani N., Heidari R., Abbaspour N., Rahmani F. (2014). Evaluation of salinity effects on ionic balance and compatible solute contents in nine grape (*Vitis* L.) genotypes. J. Plant Nutr..

[16] Hernandez-Orte P., Guitart A., Cacho J. (1999). Changes in the concentration of amino acids during the ripening of *Vitis vinifera* Tempranillo variety from the Denomination d'Origine Somontano (Spain). Am. J. Enol. Vitic..

[17] Jogaiah S., Oulkar D.P., Banerjee K., Raveendran P., Rokade N.D. (2010). Amino acid composition of major table and wine grape cultivars growing under semiarid climate in India. Hortic. Environ. Biotechnol..

[18] Sefton M.A. (1998). Hydrolytically-released volatile secondary metabolites from a juice sample of *Vitis vinifera* grape cvs. Merlot and Cabernet Sauvignon. Aust. J. Grape Wine Res..

[19] Hayasaka Y., Parker M., Baldock G.A., Pardon K.H., Black C.A., Jeffery D.W., Herderich M.J. (2013). Assessing the impact of smoke exposure in grapes: Development and validation of a HPLC-MS/MS method for quantitative analysis of smoke-derived phenolic glycosides in grapes and wine. J. Agric. Food Chem..

[20] Ferrerira V., Lopez R., Cacho J.F. (2000). Quantitative determination of the odorants of young red wines from different grape varieties. J. Sci. Food Agric..

[21] Czerny M., Christlbauer M., Christlbauer M., Fischer A., Granvogl M., Hammer M., Hartl C., Hernandez N.M., Scheiberle P. (2008). Re-investigation on odour thresholds of key food aroma compounds and development of an aroma language based on odour qualities of defined aqueous odorant solutions. Eur. Food Res. Technol..

[22] Burdock G.A. (2002). Fenaroli’s Handbook of Flavor Ingredients.

[23] Jounela-Eriksson P., Lehtonen M., Charalambous G. (1981). Phenols in the aroma of distilled beverages. The Quality of Foods and Beverages.

[24] Parker M., Osidacz P., Baldock G.A., Hayasaka Y., Black C.A., Pardon K.H., Jeffery D.W., Geue J.P., Herderich M.J., Francis I.L. (2012). Contribution of several volatile phenols and their glycoconjugates to smoke-related sensory properties of red wine. J. Agric. Food Chem..

[25] Mayr C.M., Parker M., Baldock G.A., Black C.A., Pardon K.H., Williamson P.O., Herderich M.J., Francis I.L. (2014). Determination of the importance of in-mouth release of volatile phenol glycoconjugates to the flavor of smoke-tainted wines. J. Agric. Food Chem..

[26] Boss P.K., Bottcher C., Davies C. (2014). Various influences of harvest date and fruit sugar content on different wine flavor and aroma compounds. Am. J. Enol. Vitic..

[27] Smyth H.E. (2005). The Compositional Basis of the Aroma of Riesling and Unwooded Chardonnay Wine. Ph.D. Thesis.

[28] Keyzers R.A., Boss P.K. (2010). Changes in the volatile compound production of fermentations made from musts with increasing grape content. J. Agric. Food Chem..

[29] Dennis E.G., Keyzers R.A., Kalua C.M., Maffei S.M., Nicholson E.L., Boss P.K. (2012). Grape contribution to wine aroma: Production of hexyl acetate, octyl acetate, and benzyl acetate during yeast fermentation is dependent upon precursors in the must. J. Agric. Food Chem..

[30] Iland P.G., Bruer N., Edwards G., Weeks S., Wilkes E. (2004). Chemical Analysis of Grapes and Wine: Techniques and Concepts.

[31] Long D., Wilkinson K.L., Poole K., Taylor D.K., Warren T., Astorga A.M., Jiranek V. (2012). Rapid method for proline determination in grape juice and wine. J. Agric. Food Chem..

[32] Pollnitz A.P., Pardon K.H., Sykes M., Sefton M.A. (2004). The effects of sample preparation and gas chromatograph injection techniques on the accuracy of measuring guaiacol, 4-methylguaiacol and other volatile oak compounds in oak extracts by stable isotope dilution analyses. J. Agric. Food Chem..

[33] Forde C.G., Cox A., Williams E.R., Boss P.K. (2011). Associations between the sensory attributes and volatile composition of Cabernet Sauvignon wines and the volatile composition of the grapes used for their production. J. Agric. Food Chem..

[34] Lawless H.T., Heymann H., Heldman D.R. (2010). Descriptive analysis. Sensory Evaluation of Food: Principles and Practices.

