# Supplementary material for: Influence of Fruit Maturity at Harvest on the Intensity of Smoke Taint in Wine
Source: Molecules. 2015 May 18;20(5):8913–27. doi: 10.3390/molecules20058913 (PMC6272140; doi:10.3390/molecules20058913)
Supplement: Supplementary file 1 [file molecules-20-08913-s001.pdf]

Supplementary

Table S1. Mean ratings for sensory attributes of control and smoke-affected Chardonnay, Sauvignon Blanc, Merlot and Shiraz wines.

	**Chardonnay**					**Sauvignon Blanc**					**Merlot**					**Shiraz**				
	**Harvest A**		**Harvest B**		***P***	**Harvest A**		**Harvest B**		***P***	**Harvest A**		**Harvest B**		***P***	**Harvest A**		**Harvest B**		***P***
	**C**	**S**	**C**	**S**		**C**	**S**	**C**	**S**		**C**	**S**	**C**	**S**		**C**	**S**	**C**	**S**	
Aroma																				
Fruit	4.0^{ab}	3.9^{ab}	4.5^{a}	3.3^{b}	0.05	3.7^{b}	3.5^{b}	5.2^{a}	4.6^{a}	0.001	4.7^{a}	3.3^{b}	4.5^{a}	3.6^{b}	0.001	4.1	3.7	4.2	3.8	ns
Smoke	1.4^{b}	2.3^{b}	1.7^{b}	4.4^{a}	0.001	1.5^{c}	4.0^{a}	2.3^{b}	2.7^{b}	0.001	1.9^{c}	6.9^{a}	1.9^{c}	4.6^{b}	0.001	1.5^{c}	4.0^{a}	2.7^{b}	4.7^{a}	0.001
Cold ash	0.8^{b}	1.1^{b}	0.8^{b}	2.9^{a}	0.001	0.9^{b}	2.4^{a}	0.9^{b}	1.6^{b}	0.0001	1.3^{b}	4.4^{a}	1.0^{b}	3.5^{a}	0.001	1.0^{b}	2.5^{a}	1.9^{a}	2.6^{a}	0.001
Earthy	2.4	2.6	2.0	3.0	ns	2.8	2.6	1.8	2.5	ns	2.7	2.0	2.2	2.3	ns	2.7	2.3	2.8	3.1	ns
Medicinal	0.8	0.9	0.6	1.1	ns	0.7	1.2	0.8	1.2	ns	1.5	2.5	1.3	1.5	ns	1.1	1.6	1.4	1.6	ns
Palate																				
Fruit	3.7	3.9	3.9	3.6	ns	3.1^{bc}	3.0^{c}	4.5^{a}	4.1^{ab}	0.05	3.3^{bc}	2.8^{c}	4.6^{a}	3.7^{b}	0.001	2.5^{c}	3.6^{b}	3.7^{b}	4.6^{a}	0.001
Smoky	1.3^{b}	2.4^{b}	1.9^{b}	4.5^{a}	0.001	1.3^{c}	3.6^{a}	1.9^{c}	2.7^{b}	0.001	1.6^{b}	5.5^{a}	2.1^{b}	4.5^{a}	0.001	1.9^{c}	3.4^{b}	2.9^{b}	4.6^{a}	0.001
Ashy aftertaste	1.2^{b}	1.7^{b}	1.6^{b}	4.0^{b}	0.001	0.8^{c}	3.5^{a}	1.5^{c}	2.5^{b}	0.001	1.6^{b}	5.5^{a}	2.3^{b}	4.6^{a}	0.001	1.6^{c}	2.6^{b}	2.6^{b}	3.8^{a}	0.001
Woody aftertaste	1.0^{b}	1.1^{b}	1.3^{ab}	1.8^{a}	0.05	0.5^{b}	1.3^{a}	1.4^{a}	1.5^{a}	0.01	1.2^{c}	2.0^{ab}	1.6^{bc}	2.4^{a}	0.01	1.3^{b}	1.8^{a}	1.7^{ab}	2.0^{a}	0.05
Medicinal	0.7^{b}	0.9^{b}	0.9^{b}	1.5^{a}	0.01	0.8	1.4	1.0	1.2	ns	0.8^{b}	1.8^{a}	0.9^{b}	1.8^{a}	0.05	1.3	1.7	1.2	1.8	ns
Metallic	1.9	1.8	1.5	1.7	ns	2.1	1.9	1.4	1.3	ns	1.6	2.0	1.9	2.0	ns	1.9^{b}	2.5^{ab}	2.6^{a}	2.8^{a}	0.05
Bitter	4.1^{b}	4.8^{a}	4.1^{b}	4.5^{ab}	0.05	1.7^{b}	1.9^{b}	3.6^{a}	3.9^{a}	0.001	2.2	2.6	3.2	3.5	ns	3.0^{b}	4.1^{a}	4.4^{a}	4.7^{a}	0.001
Drying	4.1^{b}	4.8^{a}	4.1^{b}	4.5^{ab}	0.01	4.7	4.7	4.5	4.5	ns	5.0^{b}	5.4^{b}	5.6^{ab}	6.1^{a}	0.01	5.2^{c}	6.3^{b}	7.0^{ab}	7.2^{a}	0.001
Acidity	7.6^{b}	8.5^{a}	6.7^{c}	7.6^{b}	0.001	11.6^{a}	10.9^{a}	7.0^{b}	7.1^{b}	0.001	7.9	7.5	7.3	7.5	ns	6.8^{b}	7.2^{ab}	8.0^{a}	7.6^{ab}	0.05

Values represent the mean score from three wine replicates presented to 13 judges in three replicated sensory sessions. Values followed by different letters are significantly different (compared within each variety); ns, not significant; C = control; S = smoke-affected.
